# Enhancing oral PrEP uptake among adolescent girls and young women in Africa

**DOI:** 10.1002/jia2.25876

**Published:** 2022-01-20

**Authors:** Craig J. Heck, Quarraisha Abdool Karim

**Affiliations:** ^1^ Department of Epidemiology Columbia University Mailman School of Public Health New York New York USA; ^2^ Centre for the AIDS Programme of Research in South Africa (CAPRISA) University of KwaZulu‐Natal Durban South Africa

1

In sub‐Saharan Africa, adolescent girls and young women (AGYW, aged 15–24 years) bear a disproportionate burden of HIV infection, as they are more than twice as likely to acquire HIV than their male counterparts [1]. Fortunately, oral pre‐exposure prophylaxis (PrEP) is a self‐initiated option to reduce AGYW's HIV risk; however, oral PrEP uptake remains low among AYGW. Perceived stigma and low risk perception are some reasons that preclude AGYW from seeking out oral PrEP [2, 3]. AGYW who wish to use oral PrEP understand their risk and want to protect themselves from HIV acquisition; despite their readiness and willingness, implementation factors can delay oral PrEP initiation and affect uptake. Thus, we posit that the pre‐requirement of a normal creatinine clearance (CrCl) result is an important barrier to oral PrEP uptake among AGYW. Since renal dysfunction is rare among this young demographic, we recommend immediate oral PrEP initiation, with confirmation of a normal CrCl result occurring prior to the 1‐month follow‐up visit.

Currently, the World Health Organization recommends that oral PrEP prescribers ensure normal renal function, usually done by collecting a blood or urine specimen to evaluate CrCl levels, with ≥60 ml/minute regarded as a proxy for normality [4]. In most settings, CrCl levels for men and women (aged <40 years) range from 107 to 139 and 87 to 107 ml/minute, respectively [5]. Given these ranges, renal dysfunction is considered a relatively rare condition among AGYW [6]. Recent evidence from HPTN 082, a randomized trial in South Africa to understand AGYW's oral PrEP use and improve adherence and persistence, supports this point, as no screened individuals were deemed ineligible due to renal dysfunction [7]. Evidence from Kenya, where immediate oral PrEP initiation is the standard of care, also underscores the rarity of renal dysfunction among AGYW. When examining a point‐of‐care creatinine test, the PrIYA program found that of the 3% (122/4169) of women with an estimated CrCl <60 ml/minute, the median age was 25 (IQR: 23–30) [8]. With renal dysfunction being rare among AGYW, the likelihood of contraindication is low, suggesting that confirmation of a normal CrCl result should not preclude immediate oral PrEP initiation.

In addition to CrCl levels at initiation, there is concern that oral PrEP use could worsen kidney function over time. While this may be a valid consideration, oral PrEP discontinuation due to CrCl changes is uncommon among AGYW. By tracking oral PrEP use for 12 weeks via direct observation, a clinical study in Malawi, South Africa, Uganda and Zimbabwe reported that no abnormal CrCl changes were attributable to oral PrEP use among pregnant and postpartum AGYW [9]. In HPTN 082, though some respondents temporarily discontinued oral PrEP due to conservative renal monitoring criteria (i.e. >10% decrease from baseline), none of their CrCl levels went below 60 ml/minute, and only 3 of 427 oral PrEP users permanently discontinued the intervention due to clinically relevant CrCl changes [7]. To date, there does not appear to be an association between oral PrEP use and clinically relevant renal dysfunction, largely because CrCl reductions reverse once oral PrEP is discontinued [10]. In the unlikely circumstance that an AGYW has undiagnosed renal issues and initiates oral PrEP immediately, the decrease in kidney function between initiation and the 1‐month follow‐up visit should be negligible and reversible [10]. These modest changes to creatinine levels, coupled with the rarity of renal dysfunction, underscore the safety of immediate oral PrEP initiation among AGYW. The minute decreases to creatinine also generate questions about the frequency of creatinine monitoring and other laboratory testing among long‐term oral PrEP users. Since few AGYW experience PrEP‐related renal dysfunction, creatinine testing could possibly occur more infrequently. Further evaluations of the optimal frequency of creatinine monitoring are needed. The regularity of bacterial STI and HIV testing among long‐term oral PrEP users also warrants additional investigation.

Given the low likelihood of renal dysfunction and deterioration among AGYW, the requirement of CrCl for oral PrEP initiation creates an unnecessary operational delay between the user seeking out and being offered oral PrEP. Depending on local infrastructure and personnel capacity, the time needed to collect specimens, process tests and communicate CrCl results can vary between a few days to 1–2 weeks. This lag time creates an operational quandary, as potential users’ motivation is highest at first contact and wanes over time [11]. Since it is difficult to document why individuals leave studies prematurely, we turn to an imperfect proxy measure to capture loss of motivation due to CrCl delays: study attrition between screening and enrolment. For HPTN 082, the inability to enrol within 45 days of screening was the most cited reason for attrition [7]. In FEM‐PrEP, after testing HIV positive and medical reasons, not returning for enrolment was the most common reason for post‐screening loss to follow up [12]. Relatedly, one in five potential participants were excluded from the VOICE trial because they did not complete screening and enrolment within the allotted timeframe [13]. While there are multiple factors that influence the decision to initiate oral PrEP, these attrition rates highlight the missed opportunities caused by delayed PrEP provision. Though, low oral PrEP uptake due to delayed provision is not an insurmountable issue, for enhancements to the delivery of ART and HIV testing support that immediate provision improves uptake and retention [14, 15].

Motivation to use HIV prevention methods and technologies is highest at first contact, yet CrCl, a pre‐requisite for oral PrEP initiation in most settings, creates a delay that may decrease interest over time, affecting oral PrEP uptake. To maximize potential users’ interest and improve oral PrEP outcomes, streamlined delivery is key. Therefore, for AGYW—whose HIV risk outweighs their risk of renal dysfunction—we propose immediate oral PrEP initiation, unless there is a history of renal dysfunction, as a safe and efficient approach for oral PrEP service delivery.

## FUNDING

Research reported in this publication was supported by the National Institute of Allergy and Infectious Diseases of the National Institutes of Health under Award Number T32AI114398.

## COMPETING INTERESTS

The authors declare no competing interests.

## DISCLAIMER

The content is solely the responsibility of the authors and does not necessarily represent the official views of the National Institutes of Health.

## Data Availability

CH and QAK conceptualized, wrote and edited this manuscript. Both authors reviewed and approved the final manuscript.

## References

[1] Joint United Nations Programme on HIV/AIDS . We've got the power: women, adolescent girls and the HIV response. UNAIDS; 2020.

[2] Camlin CS , Koss CA , Getahun M , Owino L , Itiakorit H , Akatukwasa C , et al. Understanding demand for PrEP and early experiences of PrEP use among young adults in rural Kenya and Uganda: a qualitative study. AIDS Behav. 2020;24(7):1–14. 31955361 10.1007/s10461-020-02780-x PMC7909847

[3] Velloza J , Khoza N , Scorgie F , Chitukuta M , Mutero P , Mutiti K , et al. The influence of HIV‐related stigma on PrEP disclosure and adherence among adolescent girls and young women in HPTN 082: a qualitative study. J Int AIDS Soc. 2020;23(3):e25463. 32144874 10.1002/jia2.25463 PMC7060297

[4] World Health Organization. WHO implementation tool for pre‐exposure prophylaxis (PrEP) of HIV infection. Module 1: clinical. World Health Organization; 2017.

[5] Pagana KD , Pagana TJ . Mosby's diagnostic and laboratory test reference‐e‐book. Elsevier Health Sciences; 2012.

[6] Hill NR , Fatoba ST , Oke JL , Hirst JA , O'Callaghan CA , Lasserson DS , et al. Global prevalence of chronic kidney disease–a systematic review and meta‐analysis. PLoS One. 2016;11(7):e0158765. 27383068 10.1371/journal.pone.0158765 PMC4934905

[7] Celum C , Hosek S , Tsholwana M , Kassim S , Mukaka S , Dye BJ , et al. PrEP uptake, persistence, adherence, and effect of retrospective drug level feedback on PrEP adherence among young women in southern Africa: results from HPTN 082, a randomized controlled trial. PLoS Med. 2021;18(6):e1003670. 34143779 10.1371/journal.pmed.1003670 PMC8253429

[8] Abuna F , Kinuthia J , Lagat H , Mugwanya KK , Dettinger J , Begnel ER , et al. A field evaluation of point‐of‐care creatinine testing within a large PrEP implementation program in Western Kenya. J Acquire Immune Defic Syndr. 2019;82(1):e8–10. 10.1097/QAI.0000000000002109 31219932

[9] Stranix‐Chibanda L , Anderson PL , Kacanek D , Hosek S , Huang S , Nematadzira TG , et al. Tenofovir diphosphate concentrations in dried blood spots from pregnant and postpartum adolescent and young women receiving daily observed pre‐exposure prophylaxis in sub‐Saharan Africa. Clin Infect Dis. 2020;73(7):e1893–1900. 10.1093/cid/ciaa1872 PMC8492211 33341883

[10] Pilkington V , Hill A , Hughes S , Nwokolo N , Pozniak A . How safe is TDF/FTC as PrEP? A systematic review and meta‐analysis of the risk of adverse events in 13 randomised trials of PrEP. J Virus Erad. 2018;4(4):215–24. 30515300 10.1016/S2055-6640(20)30312-5 PMC6248833

[11] Touré‐Tillery M , Fishbach A . The course of motivation. J Consum Psychol. 2011;21(4):414–23.

[12] Van Damme L , Corneli A , Ahmed K , Agot K , Lombaard J , Kapiga S , et al. Preexposure prophylaxis for HIV infection among African women. N Engl J Med. 2012;367(5):411–22. 22784040 10.1056/NEJMoa1202614 PMC3687217

[13] Marrazzo JM , Ramjee G , Richardson BA , Gomez K , Mgodi N , Nair G , et al. Tenofovir‐based preexposure prophylaxis for HIV infection among African women. N Engl J Med. 2015;372(6):509–18. 25651245 10.1056/NEJMoa1402269 PMC4341965

[14] Mateo‐Urdiales A , Johnson S , Smith R , Nachega JB , Eshun‐Wilson I . Rapid initiation of antiretroviral therapy for people living with HIV. Cochrane Database Syst Rev. 2019. 10.1002/14651858.CD012962.pub2 PMC6575156 31206168

[15] Pottie K , Medu O , Welch V , Dahal GP , Tyndall M , Rader T , et al. Effect of rapid HIV testing on HIV incidence and services in populations at high risk for HIV exposure: an equity‐focused systematic review. BMJ Open. 2014;4(12):e006859. 10.1136/bmjopen-2014-006859 PMC4267075 25510889

